# Effectiveness of Cucumis sativus L. Supplementation on Mild to Moderate Joint Pain: A Randomized, Double-Blind, Placebo-Controlled Study

**DOI:** 10.7759/cureus.93507

**Published:** 2025-09-29

**Authors:** Heather Hausenblas, David Hooper, Stephanie Hooper

**Affiliations:** 1 Applied Health Sciences, Jacksonville University, Jacksonville, USA; 2 Public Health, University of North Florida, Jacksonville, USA

**Keywords:** cucumis sativus l, dietary supplement, joint pain, medicinal herbs, mood

## Abstract

Background: Joint pain affects millions worldwide, impairing mobility and quality of life, and is a focus in management and therapy. Preliminary research found that the cucumber extract *Cucumis sativus L.* is safe and may have joint pain relief effects.

Objective: This study aimed to examine the effectiveness of a standardized powder (>1% idoBR1) of *Cucumis sativus L.* (Q-actin^TM^) on pain outcomes in adults with mild to moderate joint pain.

Methods: In this Consolidated Standards of Reporting Trials (CONSORT)-compliant, double-blind, placebo-controlled trial, 80 adults (mean age = 50.10) were randomized to either the* Cucumis sativus L.* group (CG; 20 mg/d) or the placebo group (PG; rice protein, 20 mg/d) for 60 days. Participants completed the Western Ontario and McMaster Universities Osteoarthritis Index (WOMAC), Lequesne Functional Index, Brief Pain Inventory, and Pain Disability Index at baseline, day 15, day 30, and day 60. Daily assessments of adherence and adverse events were also obtained.

Results: Improvements in all the pain outcomes over time for the CG were evidenced, with larger improvements in the CG compared to PG for the WOMAC, Brief Pain Inventory, and Pain Disability Index. Significant improvements in the WOMAC for the CG were evidenced on day 30 and day 60. From baseline to day 60, the CG showed individual percent changes of 31.79%, 10.07%, and 32.39% for the WOMAC, Lequesne Functional Index, and Pain Disability Index, while the PG experienced declines of 14.30%, 9.56%, and 14.96%, respectively, for the same measures.

Conclusion:* Cucumis sativus L.* shows potential as an effective herbal intervention for alleviating joint pain. Further clinical trials involving diverse populations and settings are recommended to confirm these findings.

## Introduction

Chronic inflammation is a common cause of joint pain, a widespread condition that affects millions of adults. In the United States, approximately one-third of adults report experiencing joint pain, with its prevalence increasing with age and exceeding 50% in older populations [1]. Joint pain significantly affects mobility, daily functioning, and quality of life, making it a critical area of research for developing effective therapeutic interventions and improving patient outcomes. The characteristics of joint pain, including its intensity, unpredictability, and duration, often result in significant functional limitations, work-related disabilities, disturbances in sleep, negative psychological effects (such as depression, anxiety, and stress), and an overall decline in quality of life [2-5]. Understanding the underlying mechanisms and identifying novel treatments are essential for mitigating its physical, emotional, and economic burden on individuals and society.

Recommended treatments for joint pain include exercise, supplements, physiotherapy, and pharmacological options such as nonsteroidal anti-inflammatory drugs (NSAIDs) and acetaminophen [4]. These therapies aim to alleviate pain, preserve mobility, and reduce disability. Among these, NSAIDs are the most frequently used medications for managing joint-related pain in adults [5]. Although NSAIDs can reduce pain, they offer only partial symptom relief and do not halt the progression of the underlying disease. Furthermore, their use is associated with risks, including gastrointestinal and cardiovascular complications. Overall, joint pain remains a significant health concern that is inadequately managed by current therapeutic options [6].

Consequently, the use of nutritional supplements and functional foods for joint pain management has increased, with about 50% of individuals with knee osteoarthritis reporting the use of these therapies [7-11]. Emerging research suggests that nutritional supplements may reduce joint pain and disability [9-13]. This is important considering that among those with joint pain, 80% were confident that they could manage their symptoms on their own [13]. There is a need, however, to examine the effectiveness of supplements to relieve joint pain.

In particular, *Cucumis sativus L.* (family *Cucurbitaceae*) is a vegetable widely consumed throughout the world that has therapeutic effects such as anti-inflammatory, lipid-lowering, and anti-diabetic [14-17]. For example, randomized controlled pilot trials found that *Cucumis sativus L.* (i.e., Q-actin^TM^) supplementation outperformed glucosamine-chondroitin and placebo for reducing pain in adults with moderate knee osteoarthritis [18,19]. Q-actin^TM^ is a cucumber extract with the anti-inflammatory iminosugar idoBR1 standardized to over 1%. These iminosugars can act as secondary messengers to reduce the inflammation process [20,21]. Further research in joint pain populations is needed to examine the effectiveness of *Cucumis sativus L.* in reducing pain.

The purpose of this study was to conduct an eight-week randomized, double-blind, placebo-controlled trial to examine the effectiveness of daily use of an aqueous extract of cucumber (*Cucumis sativus L.*, Q-actin^TM^) supplementation compared to placebo on pain in adults with a history (> three months) of mild to moderate joint pain. The primary outcome was joint pain, stiffness, and function, as assessed by the Western Ontario and McMaster Universities Osteoarthritis Index (WOMAC). The secondary outcomes were pain severity, impact, and adverse events.

## Materials and methods

Study design

The trial was conducted and reported in accordance with the Consolidated Standards of Reporting Trials (CONSORT) guidelines to ensure transparency and completeness in reporting randomized controlled trials [22]. This study was approved by the Sterling Institutional Review Board (IRB protocol #11651) and conducted in accordance with the ethical principles outlined in the Declaration of Helsinki. The study was also registered with ClinicalTrials.gov (i.e., https://clinicaltrials.gov/study/NCT06246383). This study was conducted in a double-blind, parallel treatment, randomized, placebo-controlled manner. Participants were randomized to the intervention and placebo control groups in a 1:1 ratio. Participants were automatically assigned to their respective groups by a computer program upon enrollment. This ensured complete allocation concealment by preventing researchers and participants from knowing which group they would be assigned to prior to enrollment, thereby minimizing selection bias. A blinded research assistant generated the random allocation sequence, enrolled participants, and assigned participants to the intervention. The independent variable was the supplement group of *Cucumis sativus L.* dietary supplementation or placebo. The dependent variables were joint pain (primary outcome) and severity of pain and its impact on functioning, the impact of pain on a person's ability to participate in everyday activities, and adverse events (secondary outcomes). A medium effect size was assumed based on previous research evaluating the effects of *Cucumis sativus L.* on pain [18,19]. Sample size power calculation indicated that 35 participants were needed in each group to achieve a power of 80% and alpha <0.05.

Participants

Participants were 81 healthy adults (men = 27 and women = 54, mean age = 50.1) with mild to moderate joint pain.

Inclusion criteria

Adults aged 30 to 70 years with a self-reported history of joint pain lasting more than three months in the knees, hips, ankles, shoulders, or hands were selected, as they represent a population most likely to benefit from a dietary supplement aimed at relieving joint pain [4]. A minimum symptom severity threshold was established by requiring a WOMAC pain index score of at least two points [4].

Exclusion criteria

Individuals meeting any of the following criteria were excluded from participation: (1) serious medical conditions (e.g., cancer, severe rheumatoid arthritis, recent heart attack or stroke, congestive heart failure, kidney disease, or other conditions that could hinder study participation); (2) taking other medications (e.g., arthritis medications or other anti-inflammatory drugs) or supplements for joint pain during the previous month; (3) recent stressful events within four weeks of baseline; (4) regular use of NSAIDs during the previous two weeks of baseline; (5) inability to walk for at least 6 minutes at a moderate-to-brisk pace; (6) current hormone therapy; (7) high alcohol consumption (i.e., more than 14 standard drinks per week for men or seven for women, or binge drinking within the past three months); (8) use of cigarettes or tobacco products within the past month; (9) high caffeine intake over the last month (exceeding 400 mg per day); (10) pregnancy, attempts at conception, or breastfeeding; and (11) people determined to be incompatible with the study protocol.

Procedures

Following preliminary screening, eligible participants provided Institutional Review Board-approved informed consent (Sterling IRB: 11651) prior to enrollment. Participants completed self-report questionnaires that had been psychometrically validated on day 0 (Pre), day 15, day 30, and day 60. Additionally, participants maintained a daily diary to document adherence and adverse events. The self-report surveys were completed via a secured SurveyMonkey (San Mateo, CA) link sent through email or text message. The survey took about 25 minutes to complete at each assessment. The primary outcome was a reduction in joint pain (i.e., WOMAC). The secondary outcomes were the severity of pain and its impact on functioning (i.e., Lequesne Functional Index and Brief Pain Inventory), the impact of pain on a person's ability to participate in everyday activities (i.e., Pain Disability Index), and adverse events. Participants were instructed to maintain their usual lifestyle habits and avoid introducing new exercise routines, dietary changes, or health interventions during the study. This information was self-reported. Data collection occurred from May 2024 to August 2024 and was securely stored electronically. No modifications were made to the trial outcomes after the study began; all primary and secondary outcomes were pre-specified in the trial protocol and remained unchanged throughout. Paracetamol (acetaminophen, as found in Tylenol) was permitted as a rescue medication for pain relief during the study, with its usage carefully recorded [23-25].

Intervention

A randomized, double-blind, placebo-controlled trial design was employed. Participants were randomly assigned to either the *Cucumis sativus L.* group (CG) or the placebo control group (PG) for the duration of the two-month trial. The randomization process was automated using computer-based randomization via SPSS (IBM Corp., Armonk, NY). Participants were instructed to consume (20 mg/d) the allocated substance, which was an aqueous ethanol extract of *Cucumis sativus L.*, standardized to contain more than 1% idoBR1, which is a novel type of iminosugar found in cucumbers. This iminosugar is key to Q-actin's anti-inflammatory properties and its ability to support joint health and mobility and was supplied by Gateway Health Alliances, Inc. (Fairfield, CA; https://www.ghainc.com/). The supplement is a concentrated 12:1 extract of the dried fruit, meaning that each unit of the extract contains the equivalent of 12 times the amount of the dried whole fruit. Aqueous ethanol is the extraction medium and then the extract is concentrated and dried. Specifically, the plant material was subjected to a standardized extraction process, which involved drying and pulverizing the material, followed by extraction using demineralized water at 50-60 degrees centigrade. The extract was then filtered, concentrated under reduced pressure, and then washed with 50% hydro-ethanol, and dried to produce a consistent and reproducible product. This process ensures the stability and potency of the active compounds, adhering to best practices for supplement preparation. Q-actin is Current Good Manufacturing Practice (cGMP) compliant and Kosher, HALAL, and ISO 9001:2015 certified. The placebo consisted of rice protein.

Adverse events

The supplement was well-tolerated, with no adverse event reported.

Blinding

To ensure that all subjects and researchers were unaware of the treatment assignments, Gateway Health Alliances labeled the supplement/placebo bottles as either A or B. The supplement and placebo pills were identical in color, odor, and size. The research team remained blinded to the contents of the bottles until the conclusion of the study. Immediately following the last assessment, the research team was unblinded. Subsequently, participants were unblinded and informed of their assigned condition.

Supplement information and adherence

Participants were instructed to take the capsules approximately 30 minutes prior to nighttime sleep. Adherence was tracked through a daily questionnaire that inquired about supplement intake. To enhance compliance, participants received daily text message reminders to take the supplement as directed.

Adherence

**Figure 1:**
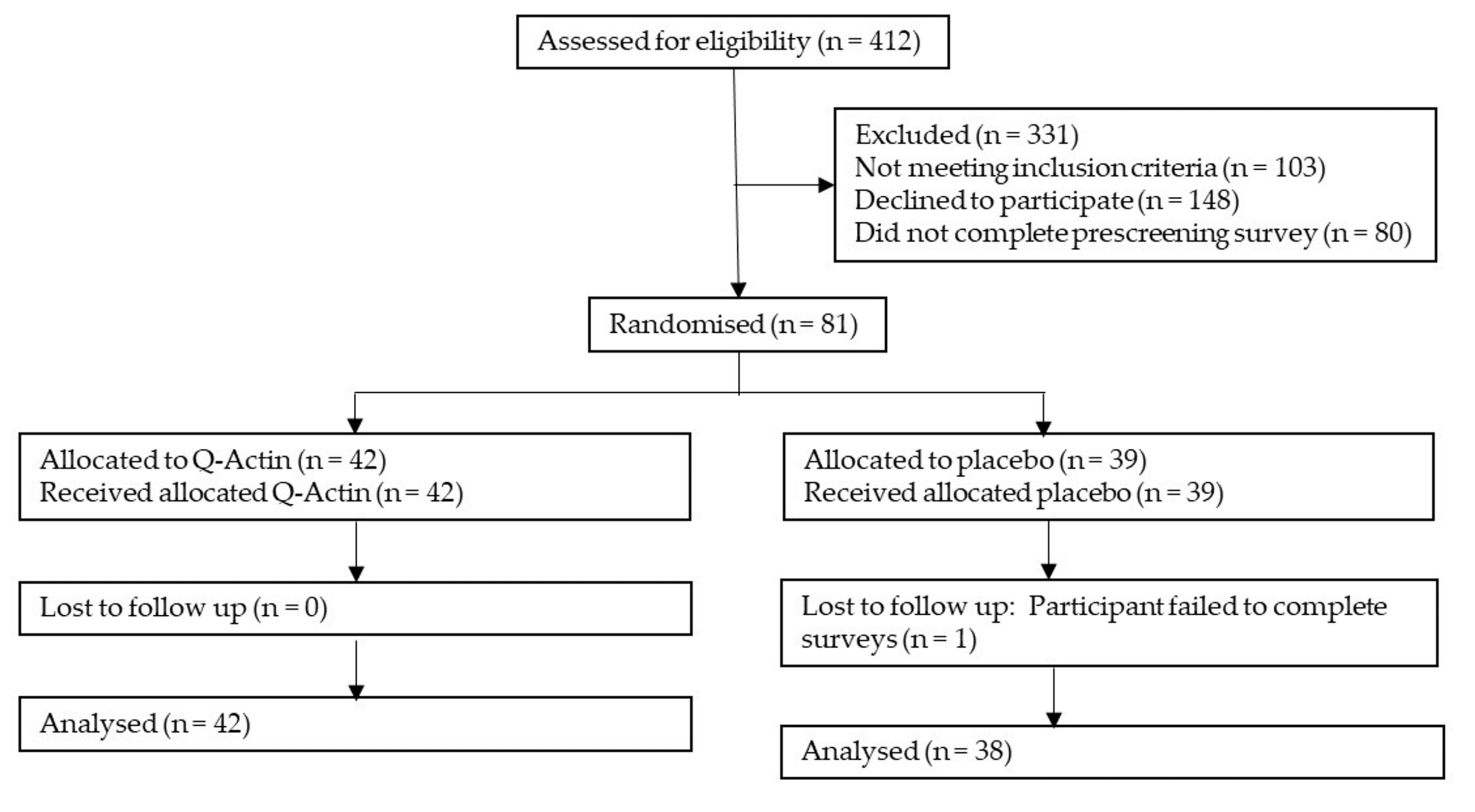
CONSORT participant flowchart. CONSORT: Consolidated Standards of Reporting Trials.

Out of 81 participants who enrolled and provided consent, 80 completed the trial, representing an adherence rate of 98.7%. This rate included one dropout from the PG who was lost to follow-up (i.e., a person failed to complete surveys) (Table 1 and Figure 1).

**Table 1:** Demographic data for the Cucumis sativus L. group and the placebo group.

Demographic variable	*Cucumis sativus L.* group (N = 42)	Placebo group (N = 38)
Age	50.10 (7.0)	50.20 (8.8)
Body mass index	27 (5.1)	26 (4.1)
Number (%), female	28, 66.7%	26, 60.5%
Ethnicity (number, %)		
White/Caucasian	38, 90%	36, 84%
Asian	1, 2%	4, 9%
Hispanic	2, 5%	2, 5%
Black/African American	1, 2%	1, 2%
Native Hawaiian or Pacific Islander	50.1 (7.0)	50.2 (8.8)

Statistical analysis

Prior to analysis, data for the outcomes were examined for normality using the Shapiro-Wilk test. For variables that showed a significant deviation from normality (p < 0.05), log10-transformed was used to reduce skewness and meet the assumptions of normality required for parametric testing. Transformed values were then used in the analyses. Results are presented using the transformed values, but back-transformed values are shown in figures and tables for interpretability. Continuous data were presented as mean (SD) and analyzed using 2 (group) x 3 (time: day 15, day 30, and day 60) repeated measures analysis of covariance (ANCOVA), with baseline values as the covariate. Post hoc tests were paired-sample t-tests where applicable. Individual percentage changes were calculated to individually capture the true variability in response across the cohort. Percent changes were calculated to represent the overall relative difference from baseline to day 60. Statistical analyses were performed using Excel (Microsoft Corporation, Redmond, WA) and SPSS version 28.

Measures

Lequesne Functional Index

The Lequesne Functional Index is a validated tool designed to assess the severity and impact of osteoarthritis on the hips or knees, primarily focusing on pain, walking distance, and activities of daily living. The Lequesne Functional Index is widely utilized in clinical research and practice to assess functional status and pain levels in patients with osteoarthritis. It enables healthcare professionals to track changes in a patient's condition over time. The index comprises multiple items that produce a composite score, representing the patient’s level of disability. Higher scores indicate more severe impairment in joint function and quality of life [26].

WOMAC

The Western Ontario and McMaster Universities Osteoarthritis Index (WOMAC™) is a tri-dimensional self-administered questionnaire to assess joint pain (five items), stiffness (two items), and physical function (16 items). The Likert version was used in this study, with five response levels for each item, representing different degrees of intensity (none, mild, moderate, severe, or extreme) that were scored from 0 to 4. Scores for the WOMAC were determined by adding item scores within each index (pain, stiffness, and function) [27].

Brief Pain Inventory

The Brief Pain Inventory is a well-established pain assessment questionnaire that has been widely utilized in both clinical and research settings to comprehensively evaluate the severity and impact of pain on daily functioning. This instrument comprises a set of questions that inquire about various aspects of pain experience, including its location, intensity, interference with activities, and the efficacy of treatments in alleviating pain. The Pain Severity Subscale consists of the following four questions that ask the patient to rate their pain: worst pain in the past 24 hours, least pain in the past 24 hours, average pain over the past 24 hours, and pain right now (current pain). The Pain Interference Score subscale consists of the following seven questions that assess how pain interferes with different aspects of the patient’s life: general activity, mood, walking ability, normal work (includes housework and job), relationships with other people, sleep, and enjoyment of life [28].

Pain Disability Index

The Pain Disability Index is a self-report tool used to evaluate the impact of pain on an individual's ability to perform daily activities and its overall effect on quality of life. Participants rate the degree to which pain limits their functional abilities, providing insight into the broader consequences of pain beyond its intensity. This measure is particularly useful for understanding how pain affects various aspects of daily living.

Daily Diary

The daily diary assessed adverse events and adherence.

## Results

Primary outcome

For the WOMAC, a significant main effect for time (F(3,231) = 8.64, p < 0.0001) was evidenced. The main effect for condition (F(1,77) = 3.44, p = 0.06) and the interaction (F(3,231) = 1.89, p = 0.13) approached significance (Table 2). The WOMAC scores decreased more over time for the CG compared to the PG, albeit nonsignificantly. Post hoc analyses indicated significant improvements in WOMAC scores from baseline to day 30 and day 60 for the CG. The individual percentage change from baseline to day 60 was 31.79% for the CG compared to a worsening of 14.30% for the PG (Table 3).

**Table 2:** Individual percentage changes from baseline scores for the Western Ontario and McMaster Universities Osteoarthritis Index (WOMAC), Lequesne Functional Index, Brief Pain Inventory (BPI), and Pain Disability Index for the Cucumis sativus L. group and the placebo group. Lower scores for all the measures indicate an improvement. * denotes a significant difference (p < 0.05) between day 0 and the different time points within the same group.

Outcome	*Cucumis sativus L.* group (N = 42)				Placebo group (N = 38)			
	Mean (SD)	Mean (SD)	Mean (SD)	Mean (SD)	Mean (SD)	Mean (SD)	Mean (SD)	Mean (SD)
	Day 0	Day 15	Day 30	Day 60	Day 0	Day 15	Day 30	Day 60
WOMAC	30.52 (14.70)	25.94 (17.19)	22.96* (14.95)	22.91* (14.66)	19.11 (11.54)	16.18 (12.88)	13.76 (10.75)	14.85 (10.90)
BPI: Pain Severity Subscale	3.66 (1.54)	3.17 (1.90)	3.05 (1.59)	3.19 (2.09)	2.50 (1.22)	2.41 (1.51)	1.99 (1.39)	2.16 (1.34)
BPI: Pain Interference Subscale	3.26 (2.03)	3.06 (2.14)	2.87 (2.13)	2.64 (2.09)	2.04 (1.81)	1.75 (1.66)	1.61 (1.85)	1.59 (1.64)
Lequesne Functional Index	9.13 (4.03)	7.16 (4.34)	7.60 (4.56)	8.37 (5.02)	7.07 (4.05)	7.37 (4.07)	5.87 (4.31)	5.82 (2.84)
Pain Disability Index	16.64 (13.50)	13.63 (13.39)	12.19 (12.22)	12.13 (12.81)	9.92 (11.04)	9.16 (11.74)	7.47 (10.43)	7.23 (9.93)

**Table 3:** Percent change (day 0 to day 60) for the Western Ontario and McMaster Universities Osteoarthritis Index (WOMAC), Lequesne Functional Index, Brief Pain Inventory (BPI), and Pain Disability Index outcomes from baseline to day 60 for the Cucumis sativus L. and the placebo control groups.

% change from baseline	*Cucumis sativus L.* group (N = 42)	Placebo group (N = 38)
WOMAC total	31.79%	-14.30%
Lequesne Functional Index	10.07%	-9.56%
Pain Disability Index	32.39%	-14.96%
Brief Pain Inventory: Pain Severity	-1.23%	6.75%
Brief Pain Inventory: Pain Interference	23.45%	59.92%

Secondary outcomes

For the Lequesne Functional Index, a significant main effect for time (F(3,231) = 8.11, p < 0.001) was evidenced. The main effect for condition (F(1,77) = 2.28, p = 0.13) and interaction (F(3,231) = 1.87, p = 0.13) was nonsignificant. Post hoc analysis indicated significantly lower scores for the PG compared to the CG at day 60 (p = 0.04). The percent change from baseline to day 60 was 10.07% for the CG compared to a worsening of 9.56% for the PG.

For the Pain Disability Index, a significant main effect for time (F(3,231) = 7.88, p = 0.001) was found. The main effect for condition (F(1,77) = 1.08, p = 0.30) and interaction (F(3,231) = 0.68, p = 0.56) was nonsignificant. The individual percentage change from baseline to day 60 was 32.39 for the CG compared to a worsening of 14.96 for the PG.

For the Brief Pain Inventory - Pain Severity Subscale, significant main effects for time (F(3,231) = 11.16, p < 0.001) and condition (F(1,77) = 3.51, p = 0.05) were evidenced. Interaction approached significance (F(3,231) = 2.31, p = 0.07). Post hoc analysis indicated significant improvements in pain severity at four weeks for the CG (p < 0.05). For the Pain Interference Score subscale, significant main effects for condition (F(1, 77) = 4.80, p = 0.02), time (F(3,231) = 11.26, p < 0.001), and interaction (F(3,231) = 2.56, p = 0.05) were evidenced. Post hoc analysis revealed greater improvements in the CG compared to the PC over time.

## Discussion

Joint pain, particularly from conditions such as osteoarthritis, significantly impacts the quality of life, reducing mobility, increasing disability risk, and contributing to mental health challenges through chronic pain. Effective management of joint pain is essential for maintaining overall physical and mental well-being, especially as joint issues become more prevalent with age. Previous research has highlighted the anti-inflammatory [14], anti-diabetic [15,16], and lipid-lowering [17] properties of *Cucumis sativus L*., and pilot trials have shown promising effects in reducing pain among adults with moderate knee osteoarthritis [18,19].

In this randomized, double-blind, placebo-controlled trial, eight weeks of supplementation with *Cucumis sativus L*. (Q-actin™) demonstrated improvements in pain and functional outcomes. Positive effects, though nonsignificant, were observed over time on the Lequesne Functional Index and Pain Disability Index. Significant effects were evidenced for the Brief Pain Inventory. These findings suggest the potential benefits of *Cucumis sativus L*. in reducing pain and enhancing function, though longer trials may be needed to detect statistically significant improvements in these measures. WOMAC scores indicated improvements in pain in both the control group (CG) and the experimental group (PG), with larger improvements noted in the experimental group. These results are consistent with previous research [17,18], which found significant reductions in pain with Q-actin supplementation, as assessed by the WOMAC.

The PG showed minor improvements, possibly due to the placebo effect or natural variability in pain perception, highlighting the importance of controlled, objective measures in assessing intervention effects. Although the placebo group’s results underscore the psychological component of pain management, the greater improvements seen with the Q-actin supplementation group indicate a potential biological benefit from *Cucumis sativus L*.

In this study, findings revealed that the CG demonstrated better scores on the Brief Pain Inventory compared to the PG, indicating that the Q-actin supplementation led to an improvement in both pain severity and pain interference scores. This suggests that *Cucumis sativus L*. may have contributed to alleviating pain and enhancing daily functioning in participants. Notably, the reduction in pain interference scores suggests that participants in the CG experienced less disruption in their daily activities, including mood, work, and sleep, compared to those in the PG. These improvements highlight the potential effectiveness of the supplements in managing pain and reducing its impact on quality of life.

The generalizability of these findings is promising but somewhat limited by the study’s sample size, homogeneous participant group, and relatively short duration. Expanding future research to include diverse populations and extended intervention periods could improve the significance and applicability of *Cucumis sativus L*. in joint health management across different demographic groups and stages of osteoarthritis. Nonetheless, this study adds to the growing evidence for *Cucumis sativus L*. as a complementary approach to managing joint pain, providing a potentially effective and safe option for individuals seeking alternative pain relief.

Over-the-counter herbal preparations for treating joint pain have limited data documenting their efficacy or safety [8-11]. Our study provides much-needed data indicating the safety and effectiveness of this supplement in adults with joint pain. Consistent with animal and lab tests [15-17], *Cucumis sativus L*. was found to be safe and well-tolerated by participants. No adverse events were reported. Further research is needed in a variety of populations (e.g., varying ages and disease risk) to establish the generalizability of these results in both clinical and nonclinical populations.

Herbal supplements are gaining prominence in contemporary healthcare due to their potential to enhance overall health outcomes, reflecting a broader trend toward integrative and complementary medicine [27,28]. To fully realize the benefits of herbal supplements, rigorous clinical research is essential to establish standardized dosages, safety profiles, and long-term efficacy. Integrating these supplements into broader healthcare strategies could provide more personalized, patient-centered care, enhancing both preventive and therapeutic outcomes.

This study has limitations that should be considered. The 60-day duration limits our ability to assess the long-term effects of *Cucumis sativus L*. on joint pain outcomes, necessitating future studies with extended follow-ups. The sample size, though sufficient for the study’s primary outcomes, may have been inadequate for subgroup analyses, and the reliance on self-reported measures introduces potential biases. Additionally, the study's findings are specific to adults with mild to moderate joint pain, limiting generalizability to more diverse populations. Further studies examining the covarying effects of anthropometric, nutritional, and socio-cultural variables are needed. While *Cucumis sativus L*. was well-tolerated, more comprehensive safety assessments, especially regarding long-term use and interactions with other treatments, are needed to further establish its safety profile.

## Conclusions

In summary, this study examined the efficacy of *Cucumis sativus L*. on joint pain in adults in a double-blind, placebo-controlled trial. The results revealed that *Cucumis sativus L*. supplementation led to reductions in pain outcomes. These preliminary findings suggest the potential of *Cucumis sativus L*. as a beneficial supplement for improving joint pain. *Cucumis sativus* extract could serve as an effective alternative to synthetic antioxidants and anti-inflammatory drugs currently available. Further research with larger sample sizes is needed for a comprehensive understanding of its mechanisms and long-term effects.

## Appendices

Questionnaires

WOMAC Questionnaire Items

1. Pain subscale (five items):

• Pain during walking on a flat surface.

• Pain during going up or down stairs.

• Pain at night while in bed.

• Pain while sitting or lying.

• Pain while standing upright.

2. Stiffness subscale (two items):

• Stiffness after first waking in the morning.

• Stiffness later in the day.

3. Physical function subscale (17 items):

• Descending stairs.

• Ascending stairs.

• Rising from sitting.

• Standing.

• Bending to the floor.

• Walking on a flat surface.

• Getting in or out of a car.

• Going shopping.

• Putting on socks or stockings.

• Rising from bed.

• Taking off socks or stockings.

• Lying in bed.

• Getting in or out of the bath.

• Sitting.

• Getting on or off the toilet.

• Heavy domestic duties.

• Light domestic duties.

Total scoring: Calculate scores for each subscale separately and sum them for a total score. Higher scores indicate worse symptoms.

Lequesne Functional Index

Lequesne Index Questions and Scoring:

1. Pain or discomfort (five items):

• Pain during walking.

0 = None

0.5 = Slight

1 = Moderate

1.5 = Severe

2 = Extreme

• Pain while standing.

0 = None

0.5 = Slight

1 = Moderate

1.5 = Severe

2 = Extreme

• Pain while sitting or lying.

0 = None

0.5 = Slight

1 = Moderate

1.5 = Severe

2 = Extreme

• Pain at night (when in bed).

0 = None

0.5 = Slight

1 = Moderate

1.5 = Severe

2 = Extreme

• Morning stiffness lasting less than 30 minutes.

0 = None

0.5 = Slight

1 = Moderate

1.5 = Severe

2 = Extreme

2. Maximum walking distance (one item):

• How far can you walk without stopping due to discomfort?

0 = Unlimited

1 = 1 km (about 0.6 miles)

2 = 500 m (about 0.3 miles)

3 = 300 m (about 0.2 miles)

4 = 100 m (about 0.06 miles)

5 = Less than 100 m (or only indoors)

3. Activities of daily living (four items):

• Difficulty getting in or out of a car.

0 = None

0.5 = Slight

1 = Moderate

1.5 = Severe

2 = Extreme

• Difficulty climbing stairs.

0 = None

0.5 = Slight

1 = Moderate

1.5 = Severe

2 = Extreme

• Difficulty squatting or bending to pick something off the floor.

0 = None

0.5 = Slight

1 = Moderate

1.5 = Severe

2 = Extreme

• Difficulty performing light domestic tasks (e.g., cleaning and cooking).

0 = None

0.5 = Slight

1 = Moderate

1.5 = Severe

2 = Extreme

Total scoring: Add up the scores for all sections.

Total score range: 0-24 (higher scores indicate greater severity).

Brief Pain Inventory (BPI)

1. Pain severity:

Purpose: Measures the intensity of pain.

Questions:

• Rate your pain at its worst in the last 24 hours.

• Rate your pain at its least in the last 24 hours.

• Rate your pain on average over the last 24 hours.

• Rate your pain right now.

Response scale: Numeric Rating Scale (0-10):

0 = No pain

10 = Pain as bad as you can imagine

2. Pain interference:

Purpose: Assesses how pain affects different areas of life.

Questions: How much has pain interfered with your:

• General activity

• Mood

• Walking ability

• Normal work (both outside the home and housework)

• Relationships with other people

• Sleep

• Enjoyment of life

Response scale: Numeric Rating Scale (0-10):

0 = Does not interfere

10 = Completely interferes

3. Pain location:

Purpose: Identifies the areas where pain is felt.

Instructions: Mark the areas where you feel pain on a body diagram.

4. Pain relief:

Purpose: Measures the effectiveness of pain relief treatments.

Question:

• In the past 24 hours, how much relief have pain treatments or medications provided?

Response scale: Percentage (0% = No relief, 100% = Complete relief).

Pain Disability Index (PDI)

The PDI consists of seven domains, each assessing how pain interferes with a specific area of life. Respondents rate the level of disability caused by pain in each domain on a scale of 0 to 10:

0 = No disability

10 = Total disability

Domains:

• Family/home responsibilities: Includes activities such as chores, home maintenance, and caring for family members.

• Recreation: Includes hobbies, sports, and other leisure activities.

• Social activity: Includes activities involving friends, social events, and community engagement.

• Occupation: Includes paid or unpaid work, school attendance, or other daily work-related responsibilities.

• Sexual behavior: Includes the frequency and quality of sexual activity.

• Self-care: Includes personal care activities such as bathing, dressing, and grooming.

• Life-support activities: Includes essential activities such as eating, sleeping, and breathing.

Instructions: Respondents are asked to consider each domain and rate how much their pain has interfered with their ability to perform activities in that area over the past week.

The total score is calculated by summing the ratings for all seven domains.

## References

[1] National Center for Health Statistics (US) (2012). Health, United States, 2011: With Special Feature on Socioeconomic Status and Health. https://pubmed.ncbi.nlm.nih.gov/22812021/.

[2] Messier SP, Loeser RF, Miller GD (2004). Exercise and dietary weight loss in overweight and obese older adults with knee osteoarthritis: the Arthritis, Diet, and Activity Promotion Trial. Arthritis Rheum.

[3] Tanamas SK, Wluka AE, Davies-Tuck M (2013). Association of weight gain with incident knee pain, stiffness, and functional difficulties: a longitudinal study. Arthritis Care Res (Hoboken).

[4] Nieman DC, Shanely RA, Luo B, Dew D, Meaney MP, Sha W (2013). A commercialized dietary supplement alleviates joint pain in community adults: a double-blind, placebo-controlled community trial. Nutr J.

[5] Breus MJ, Hooper S, Lynch T, Hausenblas HA (2024). Effectiveness of magnesium supplementation on sleep quality and mood for adults with poor sleep quality: a randomized double-blind placebo-controlled crossover pilot trial. Med Res Arch.

[6] O'Neil CK, Hanlon JT, Marcum ZA (2012). Adverse effects of analgesics commonly used by older adults with osteoarthritis: focus on non-opioid and opioid analgesics. Am J Geriatr Pharmacother.

[7] Lapane KL, Sands MR, Yang S, McAlindon TE, Eaton CB (2012). Use of complementary and alternative medicine among patients with radiographic-confirmed knee osteoarthritis. Osteoarthritis Cartilage.

[8] Sun J, Chen F, Braun C (2018). Role of curcumin in the management of pathological pain. Phytomedicine.

[9] Henrotin Y, Mobasheri A, Marty M (2012). Is there any scientific evidence for the use of glucosamine in the management of human osteoarthritis?. Arthritis Res Ther.

[10] Jerosch J (2011). Effects of glucosamine and chondroitin sulfate on cartilage metabolism in OA: outlook on other nutrient partners especially omega-3 fatty acids. Int J Rheumatol.

[11] Sawitzke AD, Shi H, Finco MF (2010). Clinical efficacy and safety of glucosamine, chondroitin sulphate, their combination, celecoxib or placebo taken to treat osteoarthritis of the knee: 2-year results from GAIT. Ann Rheum Dis.

[12] Kim N, Kim SY, Kim SW (2023). Efficacy of Perilla frutescens (L.) Britton var. frutescens extract on mild knee joint pain: a randomized controlled trial. Front Pharmacol.

[13] Oben J, Enonchong E, Kothari S, Chambliss W, Garrison R, Dolnick D (2009). Phellodendron and Citrus extracts benefit joint health in osteoarthritis patients: a pilot, double-blind, placebo-controlled study. Nutr J.

[14] Amani T, Surenthar M, Shanmugam R (2024). Anti-inflammatory and antioxidant activity of Cucumis sativus and Citrus macroptera herbal formulation: an in-vitro study. Cureus.

[15] Khan A, Mishra A, Hasan SM, Usmani A, Ubaid M, Khan N, Saidurrahman M (2022). Biological and medicinal application of Cucumis sativus Linn. - review of current status with future possibilities. J Complement Integr Med.

[16] Naureen Z, Dhuli K, Donato K (2022). Foods of the Mediterranean diet: citrus, cucumber and grape. J Prev Med Hyg.

[17] Soltani R, Hashemi M, Farazmand A, Asghari G, Heshmat-Ghahdarijani K, Kharazmkia A, Ghanadian SM (2017). Evaluation of the effects of Cucumis sativus seed extract on serum lipids in adult hyperlipidemic patients: a randomized double-blind placebo-controlled clinical trial. J Food Sci.

[18] Nash RJ, Azantsa BK, Sharp H, Shanmugham V (2018). Effectiveness of Cucumis sativus extract versus glucosamine-chondroitin in the management of moderate osteoarthritis: a randomized controlled trial. Clin Interv Aging.

[19] Nash RJ, Mafongang A, Singh H, Singwe-Ngandeu M, Penkova YB, Kaur T, Akbar J (2023). Standardised ido-BR1 cucumber extract improved parameters linked to moderate osteoarthritis in a placebo-controlled study. Curr Rheumatol Rev.

[20] Olajide OA, Iwuanyanwu VU, Banjo OW, Kato A, Penkova YB, Fleet GW, Nash RJ (2022). Iminosugar amino acid idoBR1 reduces inflammatory responses in microglia. Molecules.

[21] Kothari S, Saravana M, Muthusamy S, Mozingo A, Soni M (2018). Safety assessment of a standardized cucumber extract (Q-Actin™): oral repeat-dose toxicity and mutagenicity studies. Toxicol Rep.

[22] Schulz KF, Altman DG, Moher D (2010). CONSORT 2010 statement: updated guidelines for reporting parallel group randomised trials. BMJ.

[23] Lequesne MG, Mery C, Samson M, Gerard P (1987). Indexes of severity for osteoarthritis of the hip and knee. Validation--value in comparison with other assessment tests. Scand J Rheumatol Suppl.

[24] Bellamy N, Buchanan WW, Goldsmith CH, Campbell J, Stitt LW (1988). Validation study of WOMAC: a health status instrument for measuring clinically important patient relevant outcomes to antirheumatic drug therapy in patients with osteoarthritis of the hip or knee. J Rheumatol.

[25] Cleeland CS, Ryan KM (1994). Pain assessment: global use of the Brief Pain Inventory. Ann Acad Med Singap.

[26] Tait RC, Chibnall JT, Krause S (1990). The Pain Disability Index: psychometric properties. Pain.

[27] Hassen G, Belete G, Carrera KG (2022). Clinical implications of herbal supplements in conventional medical practice: a US perspective. Cureus.

[28] Puigdellívol Grifell J, Comellas Berenguer C, Steinbacher G (2024). Open, observational, single-arm, multicenter study assessing the effectiveness of a dietary supplement containing hydrolyzed collagen, chondroitin sulfate, and glucosamine for osteoarthritis pain reduction. J Diet Suppl.

